# Effects of undergraduate ultrasound education on cross-sectional image understanding and visual-spatial ability - a prospective study

**DOI:** 10.1186/s12909-024-05608-7

**Published:** 2024-06-05

**Authors:** Johannes Weimer, Johannes Ruppert, Thomas Vieth, Julia Weinmann-Menke, Holger Buggenhagen, Julian Künzel, Maximilian Rink, Liv Lorenz, Daniel Merkel, Carlotta Ille, Yang Yang, Lukas Müller, Roman Kloeckner, Andreas Weimer

**Affiliations:** 1 Rudolf Frey Learning Clinic, University Medical Center of the Johannes Gutenberg University Mainz, Mainz, Germany; 2 Department of Medicine, Justus Liebig University Giessen, Giessen, Germany; 3 Department of Medicine, University Medical Center of the Johannes Gutenberg University Mainz, Mainz, Germany; 4 Department of Otorhinolaryngology, Head and Neck Surgery, University Hospital Regensburg, Regensburg, Germany; 5 Department of Radiation Oncology and Radiotherapy, University Medical Center of the Johannes Gutenberg University Mainz, Mainz, Germany; 6 BIKUS—Brandenburg Institute for Clinical Ultrasound, Brandenburg Medical School Theodor Fontane (MHB), Neuruppin, Germany; 7 Department of Diagnostic and Interventional Radiology, University Medical Center of the Johannes Gutenberg University Mainz, Mainz, Germany; 8 Institute of Interventional Radiology, University Hospital Schleswig-Holstein - Campus Lübeck, Lübeck, Germany; 9 Center of Orthopedics, Trauma Surgery, and Spinal Cord Injury, Heidelberg University Hospital Heidelberg, Heidelberg, Germany

**Keywords:** Undergraduate Radiology Education, Undergraduate Ultrasound Education, Ultrasound, Visual–spatial ability, Anatomical spatial relationships, Cross-sectional image understanding, Interpretation of radiological images

## Abstract

**Introduction/aim:**

Radiological imaging is crucial in modern clinical practice and requires thorough and early training. An understanding of cross-sectional imaging is essential for effective interpretation of such imaging. This study examines the extent to which completing an undergraduate ultrasound course has positive effects on the development of visual-spatial ability, knowledge of anatomical spatial relationships, understanding of radiological cross-sectional images, and theoretical ultrasound competencies.

**Material and methods:**

This prospective observational study was conducted at a medical school with 3rd year medical students as part of a voluntary extracurricular ultrasound course. The participants completed evaluations (7-level Likert response formats and dichotomous questions “yes/no”) and theoretical tests at two time points (T1 = pre course; T2 = post course) to measure their subjective and objective cross-sectional imaging skills competencies. A questionnaire on baseline values and previous experience identified potential influencing factors.

**Results:**

A total of 141 participants were included in the study. Most participants had no previous general knowledge of ultrasound diagnostics (83%), had not yet performed a practical ultrasound examination (87%), and had not attended any courses on sonography (95%). Significant subjective and objective improvements in competencies were observed after the course, particularly in the subjective sub-area of “knowledge of anatomical spatial relationships” (*p* = 0.009). Similarly, participants showed improvements in the objective sub-areas of “theoretical ultrasound competencies” (*p* < 0.001), “radiological cross-section understanding and knowledge of anatomical spatial relationships in the abdomen” (*p* < 0.001), “visual-spatial ability in radiological cross-section images” (*p* < 0.001), and “visual-spatial ability” (*p* = 0.020).

**Conclusion:**

Ultrasound training courses can enhance the development of visual-spatial ability, knowledge of anatomical spatial relationships, radiological cross-sectional image understanding, and theoretical ultrasound competencies. Due to the reciprocal positive effects of the training, students should receive radiology training at an early stage of their studies to benefit as early as possible from the improved skills, particularly in the disciplines of anatomy and radiology.

**Supplementary Information:**

The online version contains supplementary material available at 10.1186/s12909-024-05608-7.

## Introduction

### Background

Imaging techniques such as X-ray, computer tomography (CT), magnetic resonance imaging (MRI), and ultrasound are indispensable diagnostic tools for modern medicine [1, 2]. Consequently, the number of scans performed with these imaging modalities has been continuously increasing [3, 4]. Profound and early education in these examination methods is therefore paramount in the specialty of radiology. However, it is also crucial for all physicians involved in patient care, as they must correlate the findings on imaging with the clinical presentation of patients [5–11]. Students are often now taught the basics of major radiology imaging modalities at increasing numbers of universities during their medical studies [12, 13].

Such teaching aims primarily to build competencies in interpreting radiological images of various procedures [12, 14], which develops students’ understanding of radiological and anatomical cross-sectional images [15, 16]. The basic skills required are knowledge of anatomical spatial relationships and visual-spatial ability [12, 13].

Individual universities are responsible for the implementation of the training according to study regulations. If applicable, courses should incorporate catalogues of learning objectives and recommendations from professional societies. In the context of sonography training, national competency-based learning outcomes catalogues and international professional associations suggest that sonography should be integrated into anatomy teaching during the preclinical phase to enhance understanding of anatomy. Subsequently, multiple points of contact as possible should be provided across specialties during clinical training to promote the development of practical examination skills and understanding of pathology [17–20]. These catalogues address imaging procedures in different areas of competence and disciplines and thus should be included in the training programs [13, 21]. Training approaches hence differ in timing, teaching formats, teaching methods, and scope of radiological training [11, 13, 22, 23]. Only a few non-radiological educational concepts at undergraduate level include the interpretation of cross-sectional images of anatomy [15, 16, 24]. The choice of timing and the effectiveness of teaching methods must be carefully considered so that the teaching design uses the appropriate teaching methods for each stage of study to promote skill development. In addition to teaching specific technical content, modern teaching should include general skills.

Integrating radiology training into medical studies at an early stage has various advantages. Particularly for anatomy training, radiology instruction in different imaging techniques (such as CT images, ultrasound images, MRI, or virtual anatomy training) can improve the anatomical skills of students [7, 25–29]. Films of cross-sectional images, produced by scrolling through transverse, coronal, and sagittal sections of CT and MRI scans, are advantageous in understanding anatomical spatial relationships [27, 28]. In addition, the use of ultrasound images and implementation of ultrasound training (such as with live image generation) can also be used to improve knowledge of anatomical spatial relationships as a supplement to classical anatomical dissection [26, 30–32]. Also, a high level of the core competence visual-spatial ability is crucial for the successful implementation of ultrasound-assisted punctures across various medical disciplines [33, 34]. Furthermore, there is a close relationship between high visual-spatial ability and high performance in learning anatomy [35, 36]. Ultrasound imaging is characterized by the need for the examiner to actively generate the image, correct angles and then interpret the acquired images. Depending on the angle and position of the transducer, the resulting sectional images can vary greatly and must be reorientated continuously. This leads to an active confrontation with the resulting ultrasound images, which specifically enhances the students’ spatial imagination and cognitive skills [37]. Especially this combination of practical guidance of the transducer and direct image generation can help to better understand anatomical relationships and spatial relationships [31, 37]. Ultrasound also has the advantage that it can be taught as a practical course during the degree programme whilst also being without radiation exposure, relatively quick, versatile and cost-effective compared to other imaging techniques. Students prefer a practically orientated education, so ultrasound is a highly effective way of combining theoretical and practical training.

### Research problem & aim

Overall, visual-spatial ability and an understanding of anatomical spatial relationships, anatomical cross-sections and radiological cross-sections are essential competencies required in almost all areas of medicine for the correct interpretation of radiological procedures. Several studies examine the relationships between visual-spatial ability [35, 37–40], understanding of anatomical spatial relationships [25, 27, 41–44], anatomical cross-Sects. [15, 16, 44] and radiological cross-Sects. [25, 27, 28, 42–44]. Still, only a few investigate the influence of ultrasound training on these skills [7, 37, 38]. It has already been shown that a high visual-spatial ability can improve acquisition of ultrasound skills [37, 38]. In contrast, there is evidence that visual-spatial skills can improve during an ultrasound course [37]. So far, it has only been shown in one direction that an understanding of cross-sectional radiological images can improve anatomy and visual-spatial ability [27, 28, 39]. This study aims to show whether ultrasound also enhances the other modalities to close this research gap and to clarify the correlations of these interactions. This study provides more insight into these core clinical skills and ultrasound training by examining whether and to what effect completing an ultrasound course improves visual-spatial ability, knowledge of anatomical spatial relationships and radiological cross-section image understanding.

## Methods

### Study design

**Fig. 1:**
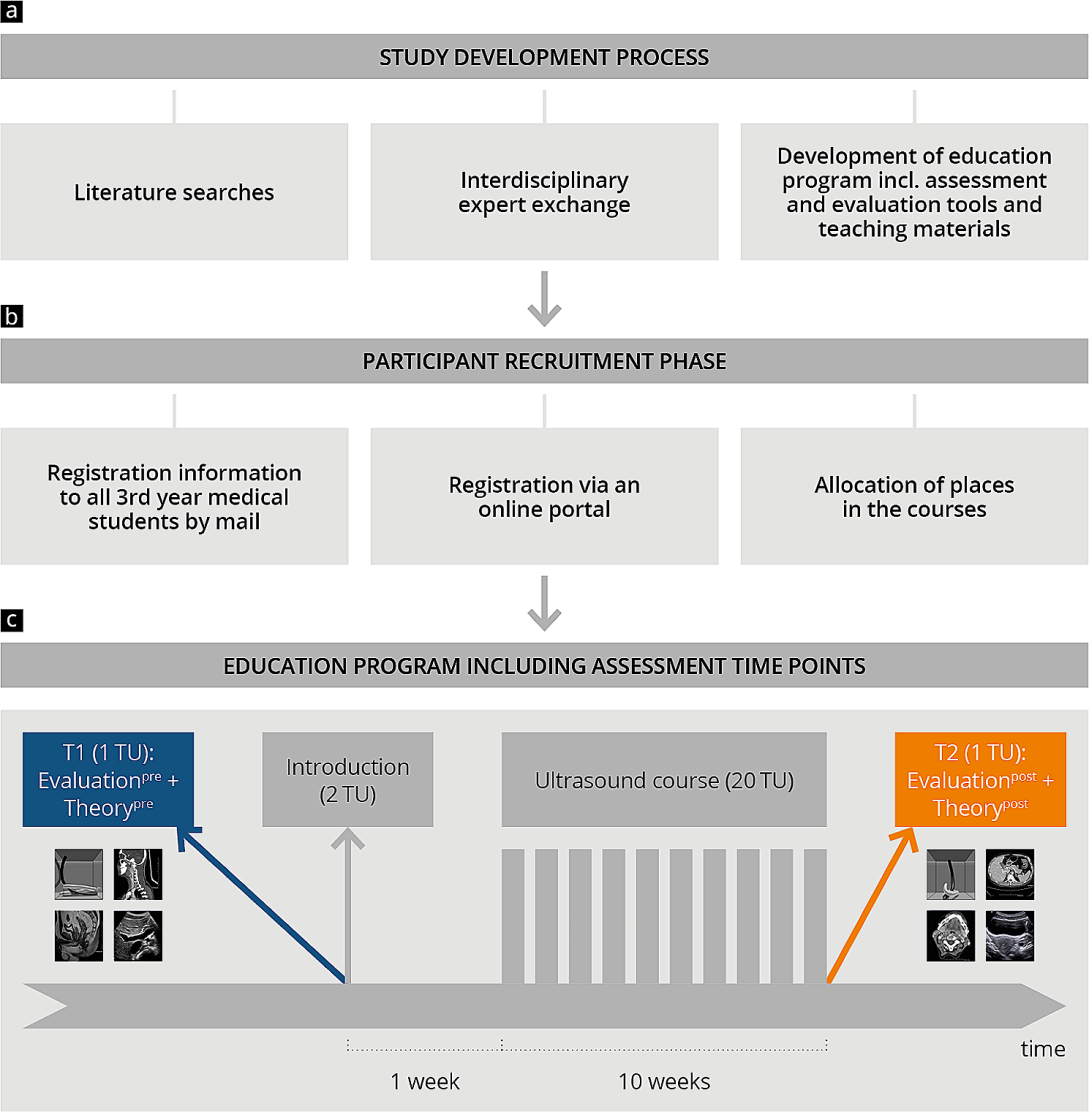
Study design including course model and evaluation time points. After the study was designed **(a)**, the participants were recruited and pooled in groups **(b)**. Participants took part in the training program and the assessment time points **(c)**. TU: Teaching unit (45 min)

This single-centre study was conducted prospectively as an observational trial at a university medical center [45]. Figure 1 outlines the protocol of the study, including data collection. The course, which was voluntary and could accommodate 160 students, was offered to all 3rd year medical students. In order to provide the earliest possible exposure to ultrasound and to include the most inexperienced users, the course was introduced during the 3rd year of study. The course included theoretical tests (Theory_pre_ and Theory_post_) and evaluations (Evaluation_pre_ and Evaluation_post_) at two time points (T_1_ = pre course; T_2_ = post course). Participants were recruited through an official advertisement sent to an e-mail distribution list from the dean’s office that included all students in their 3rd degree year. The participants who registered via an online portal were pooled in groups of 5. A total of 30 groups were taught per week over a period of 10 weeks. Inclusion criteria were passing the first state exam and participation in at least 80% of the course activities, including both theory exams and evaluations.

The primary outcome of the study is an objective improvement in visual-spatial ability, understanding of anatomical spatial relationships and radiological cross-section image understanding determined by comparison of pre- and post-tests and evaluations. The secondary outcome is a subjective increase in competence (7-level Likert response format).

### Competencies

We applied the definitions of visual-spatial ability [28, 33, 35–42, 46], 3D-Understanding [39, 46], understanding of radiological cross-Sects. [12, 44], interpretation of radiological images [12, 15, 16, 25, 27, 28, 42, 44, 47], understanding of anatomical cross-Sects. [15, 16, 27] and of anatomical spatial relationships [25, 27, 42–44] as well as theoretical and practical ultrasound competencies [7, 24, 26, 30, 31]. Table 1 summarizes the terms and their definitions as they were applied in this study.

**Table 1:** Competencies. (modified from: 7, 15, 16, 21, 23, 24, 28, 31–39, 42, 44)

Competency	Definition
Visual-spatial Ability (VSA)	Ability to interpret and mentally rotate two- and three-dimensional structures in space.
3D-Understanding	Ability to understand spatial structures and objects in three dimensions. (related to VSA)
Understanding of Radiological cross-sections (RCU)	Ability to orientate oneself in radiological sectional images (CT/MRI/ultrasound), understand the orientation, and correctly allocate structures in multiple dimensions.
Interpretation of radiological images	Ability to better understand normal physiological anatomical structures in sectional images and to recognize abnormal findings. This includes knowledge of important pathologies. (related to RCU)
Understanding of Anatomical cross-sections	Ability to orientate oneself in anatomical sectional views (on dissections or anatomical illustrations), understand the orientation, and correctly allocate structures in multiple dimensions.
Understanding of anatomical spatial relationships (ASR)	Ability to observe the three-dimensional relationships of gross anatomy and understand the relationships between anatomical structures.
Theoretical and practical ultrasound competencies (UsC)	Ability to correctly perform indicated ultrasound scan views (practical skills) and identify anatomical structures in ultrasound images (theoretical skills). Related to interpretation of radiological images.

**Fig. 2:**
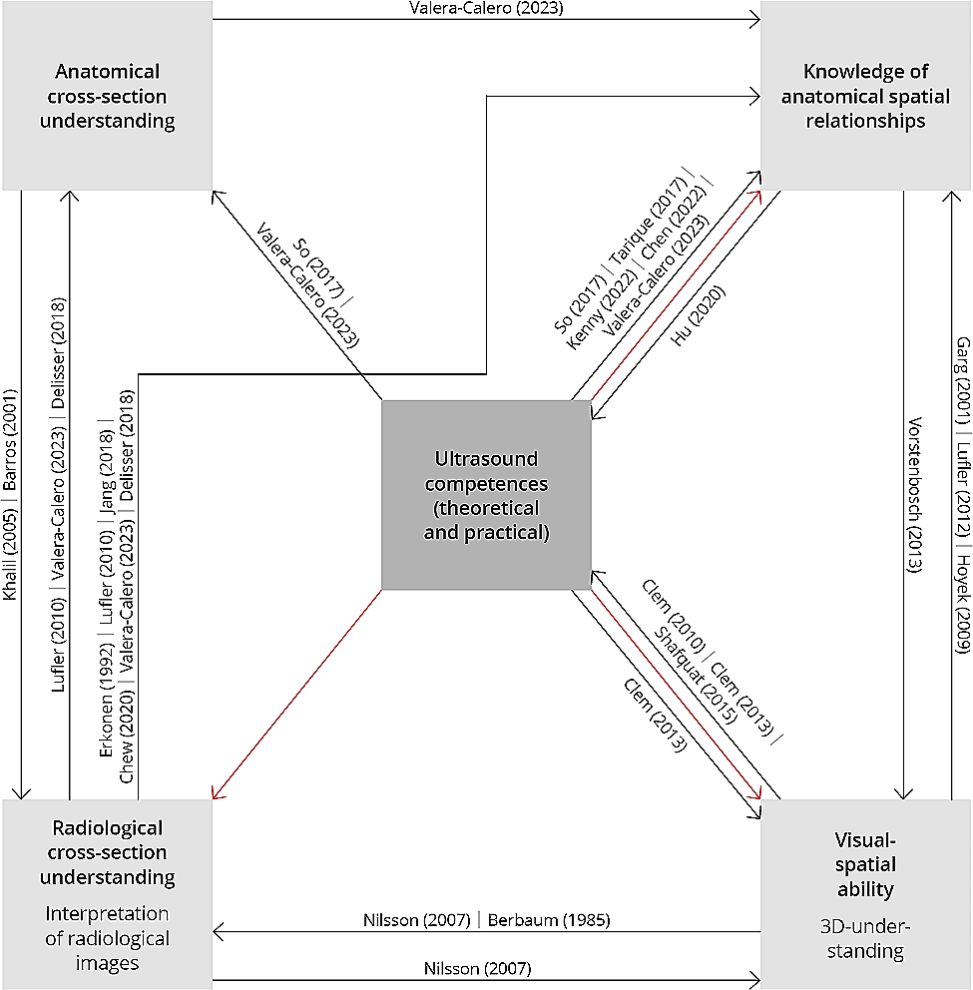
Overview of competencies and their relationships (7, 12, 15, 16, 24, 27, 30, 32, 33, 39, 42, 44, 48). Arrow (black): Influences investigated by other studies; Arrow (red): Investigated influences from our study

Figure 2 provides an overview of the relationships between these competencies based on current understanding.

### Ultrasound course

The ultrasound course (Fig. 1) was developed based on the current national resident course curricula of the German Society for Ultrasound in Medicine (DEGUM), comparable peer-to-peer concepts, and the recommendations of other professional societies [17, 18, 50–55]. The course comprises 20 German teaching units (TU) of 45 min each, for a total of 15 h, with an emphasis on abdominal sonography and some head and neck sonography (Supplement 1).

Participants voluntarily completed the Theory_pre_ test and Evaluation_pre_ questionnaire at time point T_1_ before an introduction to the course. During the introduction, the participants received information about the course and the basics of ultrasound physics. After the introduction, participants completed a 10-week course with one session of 90 min per week. The participants received lecture notes for course preparation, containing only ultrasound images and no other cross-sectional images such as MRI or CT.

All participants had the opportunity to spend the same amount of time practising with the ultrasound device. As part of the practical training, students practiced ultrasound examinations on each other. During the course, groups of 5 participants were taught by 1 peer tutor. Each session included a short review of the theoretical principles and a discussion of common pathologies with slide presentations. In the last session, the participants completed an ultrasound exam to evaluate their practical ultrasound skills as previously reported [56]. After that, at time point T_2_, they voluntarily completed the Theory_post_ test and Evaluation_post_ questionnaire.

### Questionnaires

The themes “basic characteristics”, “previous experience in general medicine”, “previous experience in radiology”, “previous experience in cross-sectional imaging”, “self-assessment”, “course preparation”, and “engagement with radiological topics during the course” were queried by dichotomous questions (“yes”/“no”), single and multiple choice questions, and 7 level Likert response formats [57].

### Theory test

The theory tests were developed based on current literature by an interdisciplinary panel of experts in radiology, internal medicine, and didactics [12, 15, 16, 27, 32, 33, 39, 48]. The test consisted of 45 multiple-choice questions with a maximum score of 45 points available. The questions in the pre-and post-test were identically worded but contained different, new images to minimize recognition bias. The images used in the test were CT and MRI images, ultrasound images, and tube figure images (see Supplement 2 for an excerpt). 40 min were available to complete each test with 40 s per Visual-Spatial Ability question and 60 s for all types of other questions. The questions and images from the test were shown as a screen presentation in the lecture hall. After the processing time for a question had expired, the next question was displayed. The participants gave their answers in writing on a sheet of paper. The test addressed the following competencies:

1. “Visual-spatial ability” (**VSA**): 15 multiple choice questions with tube figures as a modified mental rotation test modified after Vandenberg [33, 39, 40, 42, 48].
2. “Radiological cross-section image understanding (**RCU)**” + “knowledge of anatomical spatial relationships (ASR)” = (**RCU-ASR**):
  - “Visual-spatial ability in radiological cross-sections” (VSA-RC): 15 multiple choice questions with combinations of CT or MRI cross-sections and ultrasound still images. Participants had to identify anatomical features in varying cross-sections (transversal, frontal, sagittal) or had to define the orientation of different cross-sectional planes in relation to each other based on the mental rotation test [48] and radiological cross-section image understanding [12, 15, 16, 44].
  - “Understanding of radiological cross-sectional images and knowledge of anatomical spatial relationships in CT and MRI images of the abdomen and neck” (RCU-ASR-abd.) + (RCU-ASR-neck); based on preliminary works [15, 16, 32, 40], participants should identify anatomical structures in cross-sections of abdomen, pelvis and head-neck.
    i. **RCU-ASR-abd**: 7 multiple choice questions with CT and MRI cross sections.
    ii. **RCU-ASR-neck**: 3 multiple choice questions with CT and MRI cross Sect. 
  3. “Theoretical ultrasound competencies” (**UsC**): 5 multiple choice questions with still images from ultrasound; based on preliminary works [7, 24, 30, 44], participants should identify anatomical structures in sagittal and transverse sectional ultrasound images.

### Statistical analysis

Prior to the start of the study, we performed a power calculation with the following parameters: effect size of 40%, power of 90%, and significance level of 0.05. This calculation indicated that a group size of *n* = 99 would be required. The data was stored in a Microsoft Excel spreadsheet. All statistical analyses were performed in Rstudio (Rstudio Team [2020]. Rstudio: Integrated Development for R. Rstudio, PBC, http://www.rstudio.com, last accessed on 15 01 2024) with R 4.0.3 (A Language and Environment for Statistical Computing, R Foundation for Statistical Computing, http://www.R-project.org; last accessed on 15 01 2024). Binary and categorical baseline variables are given as absolute numbers and percentages. Continuous data are given as median and interquartile range (IQR) or as mean and standard deviation (SD). Categorical variables were compared using Fisher’s exact test and continuous variables using the T-test or the Mann-Whitney U test. Moreover, these tests were used to calculate the influence of the factors on the subjective and objective results. In addition, effect size was determined using Cohen’s d in a two-sample design. Parametric (ANOVA) or non-parametric (Kruskall-Wallis) analyses of variance were calculated and further explored with pairwise post hoc tests (T-test or Mann-Whitney U). Before the inference statistics, we conducted pairwise correlations of variables and plotted the correlation effect sizes and significances. P-values < 0.05 were considered statistically significant.

## Results

### Descriptive statistics and questionnaires

Out of the 220 students in the 3rd year, 145 students applied for the 160 places that were available. The statistical analysis included a total of *n* = 141 data sets. Table 2 lists the participants’ demographic details, including their reported prior experience, from Evaluation_pre_. The study group had a mean age of 25 ± 4 years, was predominantly female (66%), and most participants (77%) reported having completed prior training in the medical field. Most participants stated that they had neither general prior knowledge of ultrasound diagnostics (83%) nor had performed practical ultrasound examinations (87%) and that they had not yet attended any ultrasound courses (95%).

Most participants attended all 9 teaching sessions (8.5 ± 0.4 Sessions). The average preparation time per week was 3.05 h (± 1.2 h), of which an average of 1.3 h (± 0.8 h) was spent practising independently on the ultrasound device with the remainder dedicated to the theoretical processing of the course lecture notes. Most participants (85.7%) did not study other radiological topics such as MRI, CT, or X-rays during the course.

**Table 2:** Baseline characteristics and prior experience; *Participation in a test for medical degree programs, during which the Visual-Spatial Ability (VSA) is also assessed

Skala	Typ	Value			
Age at T_1_ in years	**mean ± SD**	24.9 ± 3.5			
Self-assessment: proficiency in Sonography	**average ± SD**	2.3 (± 1.1)			
Gender at T_1_	**group**	**male**	**female**	**n. a.**	
	*n* ** (%)**	47 (33.3)	93 (66.0)	1 (0.7)	
	**group**	**yes**	**no**	**n. a.**	
Prior training	*n* ** (%)**	109 (77.3)	30 (21.3)	2 (1.4)	
Prior university study	*n* ** (%)**	4 (2.80)	136 (96.5)	1 (0.7)	
Prior professional training	*n* ** (%)**	92 (65.2)	49 (34.8)	0	
Medical training	*n* ** (%)**	94 (66.7)	34 (24.1)	13 (9.2)	
Prior experience in Ultrasound	*n* ** (%)**	24 (17.0)	117 (83.0)	0	
Practical ultrasound experience	*n* ** (%)**	18 (12.8)	122 (86.5)	1 (0.7)	
Attendance ultrasound course	*n* ** (%)**	6 (4.30)	134 (95.0)	1 (0.7)	
Prior Experience in Radiology (CT, MRI; X-ray)	*n* ** (%)**	24 (17.0)	114 (80.9)	3 (2.1)	
Participation in “medical test”* before studies	*n* ** (%)**	77 (54.6)	63 (44.7)	1 (0.7)	
Time practical ultrasound experience	**group**	**0 h**	**1–3 h**	**3–6 h**	**n. a.**
	*n* ** (%)**	122 (86.5)	11 (7.8)	7 (5.0)	1 (0.7)

### Self-assessment

Supplement 3 presents the results of the participants’ subjective assessment of their competence regarding “Basic skills in the understanding of cross-sectional anatomy” at time points T_1_ (Evaluation_pre_) and T_2_ (Evaluation_post_). Overall, at T_1_ these were already high (> 4.0 scale points [SP]). A post-hoc test analysis for the subjective skills at T_1_ showed that only “visual perception” was significantly higher than “spatial orientation” (*p* < 0.01) and “implementation of spatial perception into task-related movements”. (*p* < 0.001). At T_2_ this tendency was no longer detectable. A subjective increase in competency was recorded in the overall score, but without statistical significance. The largest, significant increase in the competencies surveyed was achieved for ASR (*p* = 0.009).

### Theory tests

**Fig. 3:**
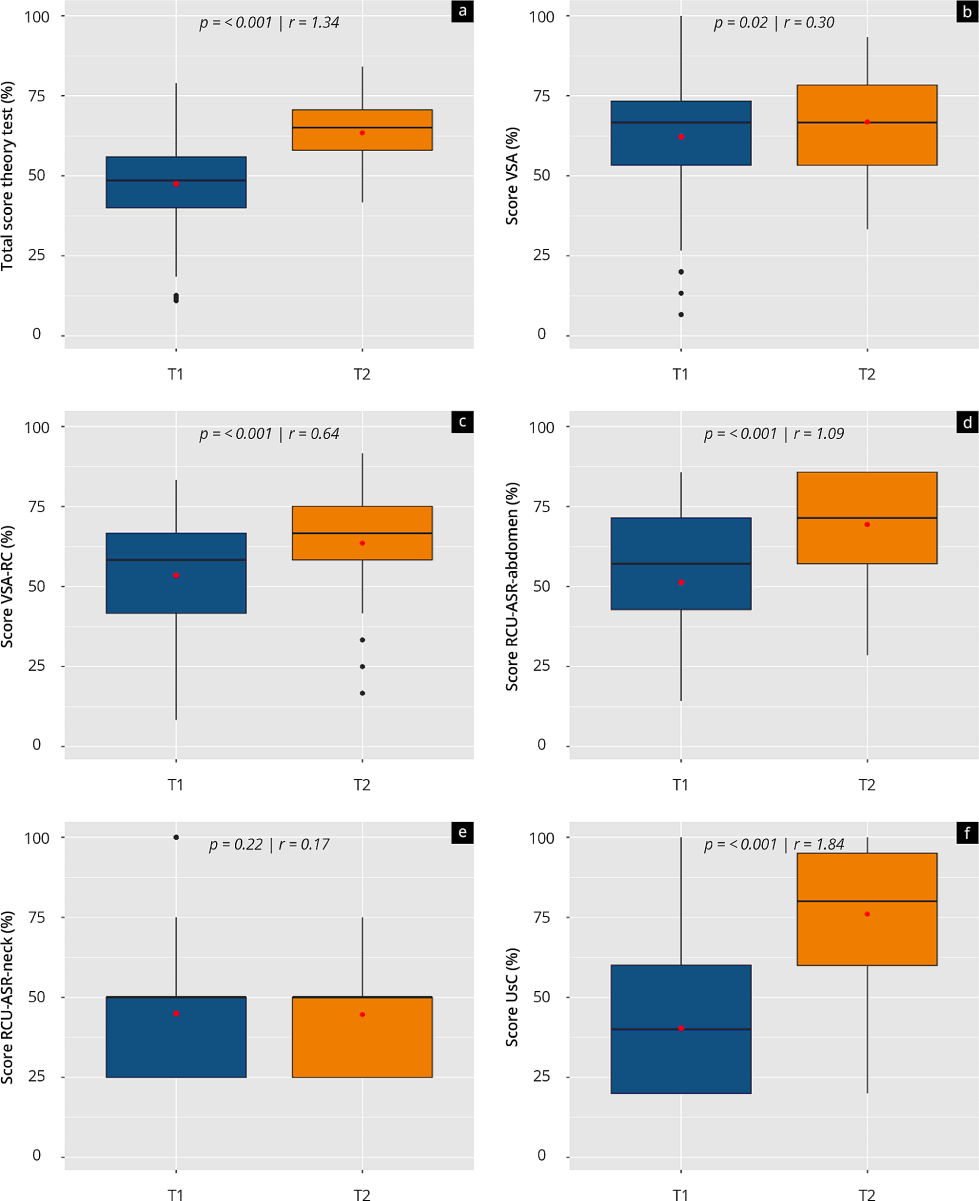
Results of the theory tests at time points T1(Theory_pre_) and T2 (Theory_post_). Box plots visualizing the respective overall score **(a)** as well as the score of the competencies: “VSA: visual-spatial ability” **(b)**, “VSA-RC: Visual-spatial ability in radiological cross-sectional images” **(c)**, “RCU-ASR- abd: Understanding of radiological cross-sectional images and knowledge of anatomical spatial relationships in CT and MRI images in the abdomen” **(d)**, “RCU-ASR-neck: Understanding of radiological cross-sectional images and knowledge of anatomical spatial relationships in CT and MRI images in the neck” **(e)**, and “UsC: theoretical ultrasound competences” **(f)**. A high number implies a high percentage performance in the test. The median (black lines), mean (red dots), and the effect size r are shown

Figure 3 and Supplement 3 show the results of the theory tests at T_1_ (Theory_pre_) and T_2_ (Theory_post_). A significant increase with a high effect size was achieved both in the overall score (*p* < 0.001) and almost all competencies tested: UsC (*p* < 0.001), RCU-ASR-abd (*p* < 0.001), VSA-RC (*p* < 0.001). and VSA (*p* = 0.02). Only RCU-ASR-neck showed no significant increase.

Both RSC-ASR-neck and theoretical UsC were initially significantly (*p* < 0.001) worse than other competencies. Significantly higher scores were initially achieved for VSA than for RSC-ASR-abd (*p* < 0.001). The same was observed for VSA-RC (*p* < 0.01).

At T_2_, participants achieved significantly (*p* < 0.001) lower scores for RSC-ASR-neck than the other competencies. UsC was completed with a significantly higher score (*p* < 0.01) than the other competencies. RSC-ASR-abd was significantly (*p* = 0.033) higher than VSA-RC.

Supplement 4 shows possible influencing factors as indicated by their correlation to the results of the theory tests at T_1_ (Theory_pre_) and T_2_ (Theory_post_). At T_1_, previous practical ultrasound experience (“yes”) had a significant correlation (*p* < 0.05) with the overall test result. “Dealing with other radiological topics” correlated to a significantly higher (*p* < 0.05) overall test result at T_2_.

The analysed correlations between the total scores of subjective assessments and objective competencies at T1 and T2 indicate that while no linear relationship was found at T1 (*R* = 0.083; *p* = 0.33), a significant positive linear relationship was observed at T2 (*R* = 0.35; *p* = 0.0031).

At both T_1_ and T_2_, the subjective competencies surveyed tended to have a weakly positive to moderately strong correlation with one another. In particular, the self-assessment of ultrasound skills correlated significantly and positively with the self-assessment of topographical understanding at T_1_ (*R* = 0.53, *p* = 0.005). In addition, a significant positive linear relationship was found between the self-assessment of topographical understanding and the objective examination performance at T_2_. The objective results of ultrasound skills/understanding correlated significantly positively with the results of the tube figures (*R* = 0.32, *p* = 0.007).

Students who participated in the “medical test before their studies” had a significantly better result in the overall test (*p* < 0.01).

## Discussion

### Summary of key results

This prospective study examined the effects of a student ultrasound course on visual-spatial ability, understanding of anatomical spatial relationships, radiological cross-sections image understanding, and theoretical ultrasound competencies. In summary, a significant objective increase in these skills was found, accompanied by an improvement in subjective skills. These increases were particularly significant for the “understanding of anatomical spatial relationships” competency.

### Interpretation of subjective and objective gain in competencies

A slight, but insignificant improvement in the subjective assessment of personal skills was observed. The high number of participants with previous training in the medical field in the study group might have skewed the results towards higher initial skill levels. The significant subjective improvement in the “knowledge of anatomical spatial relationships” illustrates the influence of ultrasound training on anatomical/topographical knowledge and could be due to a better understanding of the anatomy through practice and experiencing the anatomical structures live during the examination training [7]. The significant correlation between self-assessment of ultrasound skills and topographical understanding also reflects this aspect. For this reason, ultrasound courses should be implemented in anatomy training [26, 58].

In addition to an increase in subjective competencies, a significant improvement in objective competencies was detected, namely in visual-spatial ability (VSA), knowledge of anatomical spatial relationships (ASR), radiological cross-sectional image understanding (RCU), and theoretical ultrasound competencies (UsC). Each competency is discussed in turn below.

VSA, i.e. the ability to interpret and manipulate spatial relationships, is an essential competency in the performance of interpreting radiological images [59]. VSA has been proven to be an important factor in the acquisition of skills in sonography [37, 38]. There has been limited research into how an ultrasound course improves spatial imagination [37, 38]. Consistent with our findings, one study found a significant improvement in VSA among learners after a structured ultrasound course [37]. In contrast to our study, VSA improvement was tested using the Revised Minnesota Paper Form Board Test [37]. Though not directly comparable, our participants also exhibited a significant correlation between their ultrasound skills and the results of the tube figure test in the post-test, and while the prior study examined a total of 73 participants, we were able to find similar results in a larger cohort (i.e., medical students from an entire university semester) [37].

VSA is important in other areas of clinical learning, such as understanding anatomy. Some studies have shown that good spatial imagination correlates positively with exam performance in anatomy courses [35, 36, 40], and learning anatomy has a positive influence on spatial imagination [41]. VSA is vital in surgical procedures and interventional procedures [33, 34], including ultrasound-assisted punctures [33].

Studies often discuss gender differences in improving VSA. While some studies describe actual differences [33, 39, 42], others could not detect differences [40], as in this study. Yet if we could not replicate gender-based findings, our study is consistent with others in suggesting that VSA is not a static competency, as it improves through training [35, 40, 42]. Students with low levels of VSA can be supported through training to achieve a field-specific increase in competence [35, 40, 42], and our findings suggest that ultrasound training is one way to effect this increase.

An understanding of ASR is the knowledge of spatial relationships of macroscopic anatomy and the relationships between anatomical structures. Teaching imaging techniques (specifically X-ray, CT, MRI, and ultrasound) has been found to help learners better understand complex anatomical structures and topographical relationships [25, 27, 28, 43, 44]. Macroscopic-anatomical examination performance improves after radiology training [25, 27, 28, 43, 44]. As is consistent with prior findings, we observed a significant improvement in the identification of anatomical structures in radiological images (RCU-ASR-abd.). While we observed lower scores in the RCU-ASR-neck aspect of the objective test, this might be explained by either the relative paucity of head and neck sonographic content taught in the course, or by the more complex anatomy, or by the slightly lower quantity of questions in the exam. Regardless, ultrasound training is suitable for teaching (cross-sectional) anatomy and is advantageous for developing or deepening prior knowledge of anatomy [7]. This study confirms these results and affirms the recommendation to incorporate ultrasound when teaching anatomy.

RCU, i.e. the ability to correctly orientate oneself in radiological cross-sectional images and to correctly assign structures, is based on visual-spatial ability and knowledge of anatomical spatial relationships. A study testing depth perception in X-ray images showed that high visual-spatial ability makes it easier to understand 3D information in such images [39]. While this prior investigation used summation images, in which the illuminated structures are superimposed, rather than cross-sectional images as in our study, we agree with its finding that both VSA and other factors are important for the interpretation of 3D information in radiological images. Indeed, we echo De Barros et al. (2001) who were able to show that the interpretation of radiological cross-sections could be improved through a specific course in cross-sectional anatomy [16]. As in our study, their testing involved the assignation of anatomical structures in cross-sectional images, and through the combined presentation of anatomical and radiological cross-sections, the learners’ understanding of radiological cross-section images was improved [15, 16]. The ultrasound training in our study has a further advantage in that students receive an interactive combination of anatomical spatial relationships and the direct generation of radiological cross-sections by live ultrasound examination practice on volunteers. To our knowledge, there are currently no specific studies with ultrasound courses examining the influence of ultrasound training on understanding radiological cross-sections in detail. Yet the data from this study indicate a positive influence of ultrasound training on visual-spatial ability, understanding of anatomical spatial relationships, and understanding of radiological cross-sections that warrants further investigation. Ultrasound in practical training sessions proved to be an effective and interactive teaching tool for the training of radiological cross-section image understanding.

UsC improved significantly, suggesting that one of the main goals of the course was achieved as was the case in other studies [8, 30]. Curricular and extracurricular training for ultrasound diagnostics should be integrated into the degree program [17, 18]. In addition, contact with the radiology discipline at an early stage could increase students’ general interest in the field of radiology and even influence their choice of speciality after completing their degree [8, 46, 60].

### Summary of future perspectives and implications for ultrasound training

Training concepts for radiological sectional imaging should be combined more effectively and incorporated earlier into degree programs. Ultrasound is beneficial for developing and deepening anatomical knowledge, as well as providing further interactive clinical imaging training, facilitating an easier transition into the workplace after graduation. Additionally, students can be specifically supported through targeted assessment of their skills.

### Limitations

The tests were developed based on the current state of science and research. While VSA was assessed through a validated test [33, 39, 40, 42, 48], similar to comparable studies [15, 16, 32]), the newly developed parts of the test assessing UsC, RCU-ASR, and VSA-RC competencies have yet to be validated. The authors tried to select the same task structures with images that were equivalent in content but different, but not fundamentally different in terms of difficulty for the pre-and post-tests. Because the tests were part of a voluntary student ultrasound course, randomization into the study and control groups was not possible. Participants were acquired consecutively. Possible confounding factors, such as practical ultrasound experience or previous medical training were identified as tangible influencing factors and included in the analysis of the data. A high number of participants had previous medical training, but most reported little experience with ultrasound and the interpretation of imaging procedures. The improved results for students who took part in the “medical test before studies” can potentially be explained by the earlier intensive exploration of spatial perception tests. This connection could be investigated further in future studies, in particular whether there is an improvement in practical ultrasound skills. However, it cannot be ruled out that other personal factors (e.g. motivation) could have had a possible influence on the results. In general, the lack of a control group may affect the generalizability of the results.

## Conclusion

The study shows that participation in an ultrasound course can develop competencies in visual-spatial ability, knowledge of anatomical spatial relationships, and understanding of radiological cross-section images. Due to the mutually positive effects, students should receive radiological training at an early stage of their studies to benefit from the improved skills as early as possible. A combination of different teaching methods incorporating different cross-sectional image modalities, including ultrasound imaging, is advantageous, as the combination of practical and theoretical components enables multidimensional, dynamic learning of cross-sectional image representations. Future studies should focus on more precise correlations between the various competences and their interrelationship, also in the context of the digitalization within ultrasound training.

### Electronic supplementary material

Below is the link to the electronic supplementary material.

Supplementary Material 1

Supplementary Material 2

Supplementary Material 3

Supplementary Material 4

## Data Availability

Data cannot be shared publicly because of institutional and national data policy restrictions imposed by the Ethics committee since the data contain potentially identifying study participants’ information. Data are available upon request from the Johannes Gutenberg University Mainz Medical Center (contact via weimer@uni-mainz.de) for researchers who meet the criteria for access to confidential data (please provide the manuscript title with your enquiry).
