# Supplementary material for: Effects of undergraduate ultrasound education on cross-sectional image understanding and visual-spatial ability - a prospective study
Source: BMC Med Educ. 2024 Jun 5;24:619. doi: 10.1186/s12909-024-05608-7 (PMC11151628; doi:10.1186/s12909-024-05608-7)
Supplement: Supplementary file 1 — Supplementary Material 1 [file 12909_2024_5608_MOESM1_ESM.pdf]

Supplement 1 Learning objectives of the sonography modules of the course

Module	Learning objectives	
0 (introduction)<br>Basics	Device operation, transducer posture patient positioning documentation in two layers, basic ultrasound physics, artefacts, screen orientation, transducer types, ultrasound terminology, essential keyboard functions, ultrasound limitations	
	**Assessed normal findings (image acquisition and identification in the sagittal and transverse plane)**	**Assessed pathologies**
1 + 2a<br>Abdominal<br>Vessels	Abdominal aorta, coeliac trunk, superior mesenterial artery, renal arteries, left and right common iliac arteries, inferior vena cava, hepatic veins, splenic vein, portal vein, renal veins	Aortic plaque, aortic aneurysm, aortic dissection, vena cava inferior congestion, benign and malignant lymph nodes
2b<br>Pancreas	Pancreatic head and body, pancreatic duct, uncinate process, pancreatic tail	Acute and chronic pancreatitis, concretion in ductus pancreaticus, pancreatic lipomatosis, pancreatic carcinoma, congested ductus pancreaticus
3<br>Portal area of liver, biliary tract, gallbladder	Ductus hepatocholedochus, proper hepatic artery, portal vein, intrahepatic bile ducts, gallbladder pre- and postprandial	Portal vein dilatation, portal vein thrombosis, cholestasis, tumor in biliary duct, biliary calculus, gallstone, cholecystitis, sludge and hydrops, cholesterol polyps
4/<br>Liver	Hepatic vein star, portal vein plane, intrahepatic bile ducts, liver segments	Diffuse liver lesions (hepatic cirrhosis, Steatosis hepatis), benign and malign focal lesions, Intrahepatic cholestasis
5a/<br>Kidneys	longitudinal and transverse organ axis, kidney width and length, pyelon/parenchyma ratio, psoas muscle as lead structure “glide sign”, hepatorenal pouch of Morrison, splenorenal pouch of Koller	Form variants, angiomyolipoma, chronic renal failure, renal carcinoma, nephrolithiasis, urinary stasis, pyelonephritis, polycystic kidney
5b<br>Spleen	Longitudinal and transverse organ axis with measurements, splenic vein, pancreatic tail, volume determination	Splenomegaly with collateral, Splen accessorius, splenic infarction, splenic calcification, splenic cysts, splenic rupture, malign focal lesions

6<br>Pelvic organs	Urinary bladder including volume determination, urinary bladder jet with color doppler, prostate including measurement, seminal vesicles, uterus including measurement, ovaries, rectum, rectovesical pouch, Douglas pouch, common iliac arteries and veins	Urinary bladder sludge and coagulum, urinary retention, residual urine, chronic cystitis, urinary bladder carcinoma, ascites/free fluid Douglas-room, prostate hyperplasia, ovarian cyst, uterine myoma, intrauterine device
7<br>E-FAST	Orientation sections of the<br>E- FAST Examination, use of the M-Mode	Hemathotorax, pneumothorax, pleural effusion; fluid perihepatic, Morison's pouch, perisplenic, Collor's pouch and Douglas space; pericardial effusion.
8<br>Head-neck	Longitudinal and transverse organ axis of the thyroid gland with measurements (volume); Imaging of the floor of the mouth, sublingual glands, submandibular glands, parotid glands	Focal and diffuse pathologies of the thyroid gland, benign and malignant pathologies of the head and neck glands
9<br>Peripheral Vessels	Cervical vascular-nerve tract in longitudinal and transverse axis; measurement of intima media thickness, valsalva manoeuvre, basic color-doppler examination; Point of care examination of the leg veins	Atherosclerosis of the vessels, Neck veins congestion; Deep vein thrombosis
10<br>Practical examination	Performance of a standardized ultrasound examination of pre-defined organs/regions	
