# Supplementary material for: Effects of undergraduate ultrasound education on cross-sectional image understanding and visual-spatial ability - a prospective study
Source: BMC Med Educ. 2024 Jun 5;24:619. doi: 10.1186/s12909-024-05608-7 (PMC11151628; doi:10.1186/s12909-024-05608-7)

SUPPLEMENT 2:
EXAMPLE QUESTIONS OF THE THEORY TEST

VSA = Visual-spatial Ability

VSA-RC = Visual-spatial ability in radiological cross-section images

RCU-ASR-neck = Understanding of radiological cross-sectional images and knowledge of anatomical spatial relationships in CT and MRI images in the neck

RCU-ASR-abd = Understanding of radiological cross-sectional images and knowledge of anatomical spatial relationships in CT and MRI images in the abdomen

UsC = Theoretical ultrasound competences

Question example 1 (VSA)

In the left picture you can see a cube and the cables it contains from the front (front view). In the illustration on the right, the cube has been rotated. Please decide from which position you are viewing the cube now!

Front view

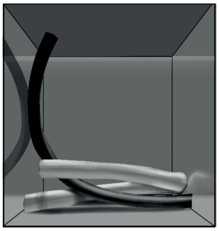

Possible answers

(A): Right
(B): Left
(C): Bottom
(D): Top
(E): Behind

Second view

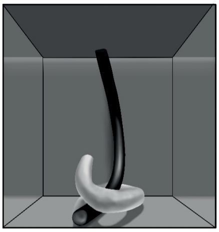

Question example VSA-RC 1

Which vessel is located **directly dorsal** to the vessel marked with the arrow?

(A): Vertebral artery
(B): External carotid artery
(C): Common carotid artery
(D): Internal carotid artery
(E): Internal jugular vein

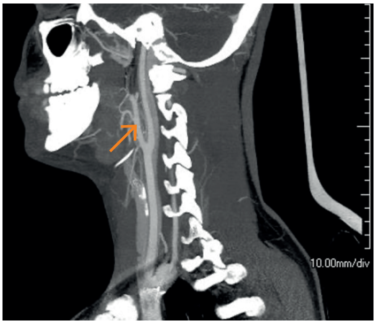

Question example VSA-RC 2

With which number is the **left side of the patient** marked?

(A): 1
(B): 2
(C): 3
(D): 4
(E): No answer is correct.

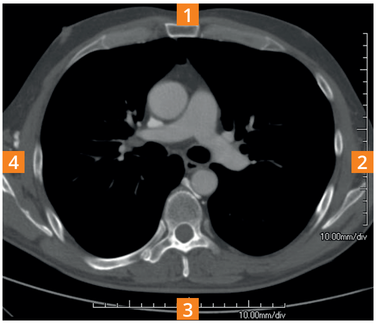

Question example VSA-RC 3

Which structure is **not located ventral** to the structure, marked with a *?

(A): Urinary bladder
(B): Symphysis pubis
(C): Prostate
(D): Rectovesical pouch
(E): Sacral bone

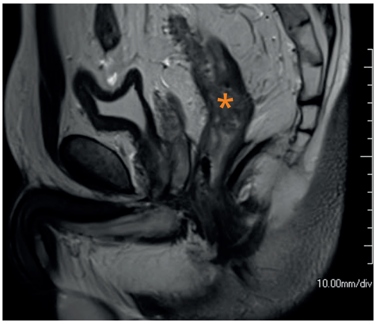

Question example VSA-RC 4

Which number / s indicate the **same structure** in the right and left images?

(A): 1 + 4 (B): 1 + 2 + 3 (C): 2 + 3 (D): 1 + 3 + 5 (E): 2

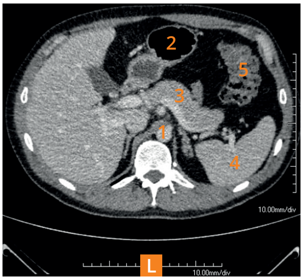

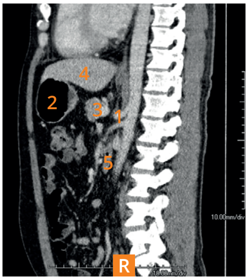

Question example VSA-RC 5

Starting from the image on the left, the image on the right was created by **rotation** of how many degrees about the origin?

(A): 90° clockwise
(B): 180° clockwise
(C): 90° counterclockwise
(D): 45° clockwise
(E): No answer is correct.

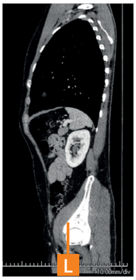

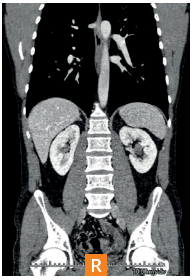

Question example VSA-RC 6

Through which of the **planes** drawn in the left image, is the **right image** created?

(A): 1 (B): 2 (C): 3 (D): 4 (E): No answer is correct.

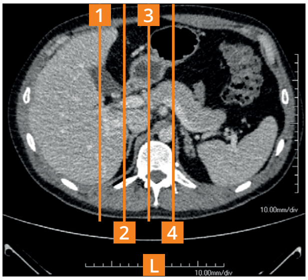

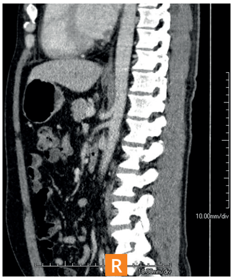

Question example VSA-RC 7

Which **numbered structure** in the right image corresponds to the structure marked with a * in the left image?

(A): 1 (B): 2 (C): 3 (D): 4 (E): No answer is correct.

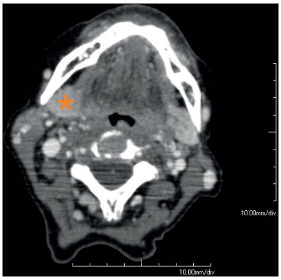

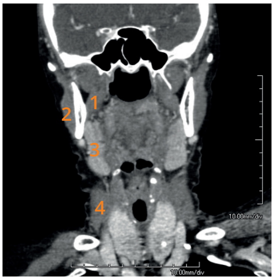

Question example VSA-RC 8

In what order are the **reference planes** displayed here?

(A): Transverse, sagittal, coronal
(B): Coronal, sagittal, transverse
(C): Transverse, coronal, sagittal
(D): Sagittal, coronal, transverse
(E): Coronal, transverse, sagittal

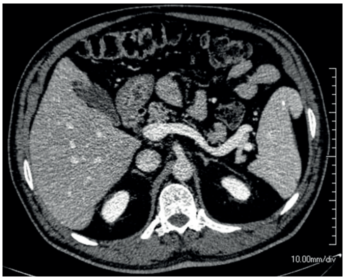

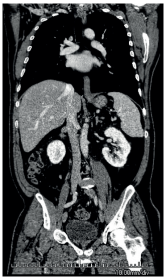

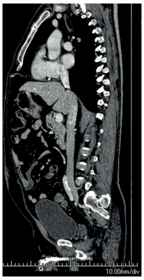

Question example VSA-RC 9

Which **number** in the right picture corresponds to the **vessel** marked with the **arrow** in the left picture?

(A): 1 (B): 2 (C): 3 (D): 4 (E): 5

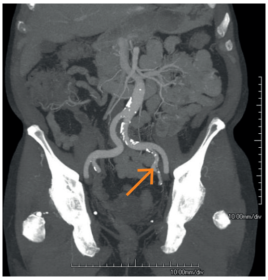

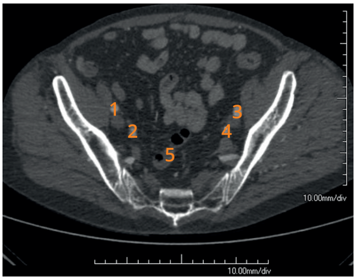

Question example RCU-ASR-neck

Which **structure** is marked with the **arrow**?

(A): Common carotid artery (B): Internal jugular vein (C): Internal carotid artery
(D): Vertebral artery (E): External carotid artery

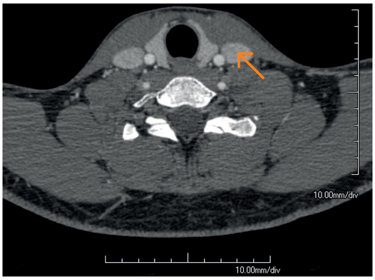

Question example RCU-ASR-abd 1

Which **structure** is marked with the **arrow**?

(A): Gallbladder
(B): Portal vein
(C): Inferior vena cava
(D): Stomach
(E): Descending Duodenum

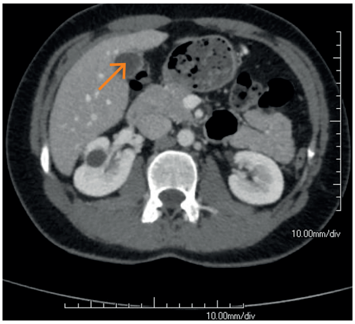

Question example RCU-ASR-abd 2

Which **number** is the **right renal vein** marked?

(A): 1
(B): 2
(C): 3
(D): 4
(E): No answer is correct.

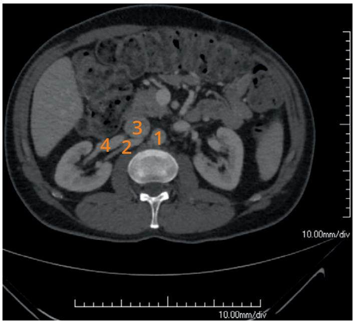

Question example RCU-ASR-abd 3

Which **number** is labeled **incorrectly**?

(A): 1 – Stomach
(B): 2 – Aorta
(C): 3 – Descending colon
(D): 4 – Right renal artery
(E): All structures are labeled correctly.

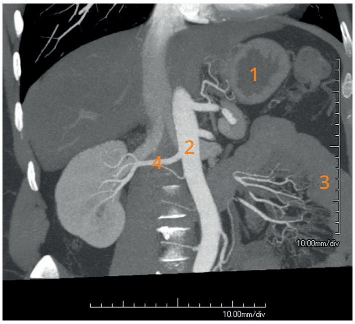

Question example UsC 1

What is the name of the **structure** that runs ventral to the inferior vena cava (IVC) and is marked with a *?

(A): Abdominal aorta
(B): Common hepatic artery
(C): Portal vein | (D): Splenic vein
(E): Superior mesenteric artery

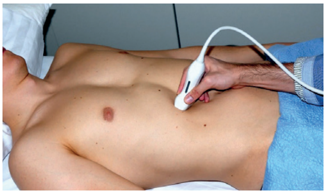

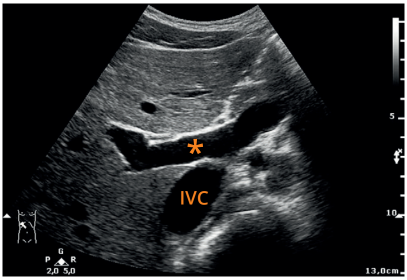

Question example UsC 2

Which **structure** is labeled **incorrectly**?

(A): The patient's left side
(B): Abdominal wall
(C): Uterus
(D): Ovary
(E): All structures are labeled correctly.

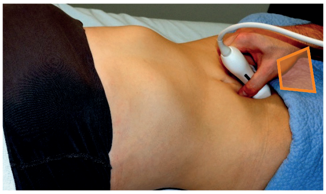

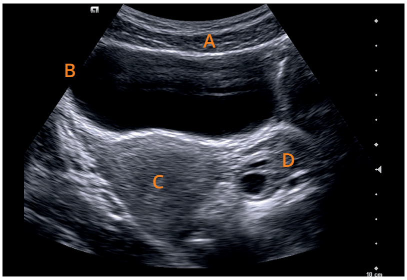

Question example UsC 3

Which **structure** is labeled **incorrectly**?

(A): Infrahyoid muscles
(B): Trachea
(C): Isthmus
(D): Left sternocleidomastoid muscle
(E): All structures are labeled correctly.

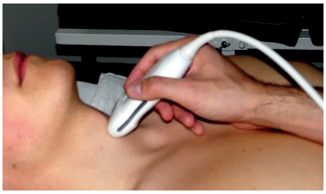

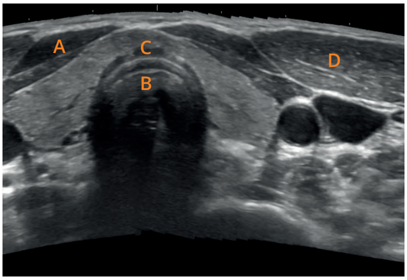

Supplement: Supplementary file 2 — Supplementary Material 2 [file 12909_2024_5608_MOESM2_ESM.pdf]
