# Supplementary material for: Effects of undergraduate ultrasound education on cross-sectional image understanding and visual-spatial ability - a prospective study
Source: BMC Med Educ. 2024 Jun 5;24:619. doi: 10.1186/s12909-024-05608-7 (PMC11151628; doi:10.1186/s12909-024-05608-7)
Supplement: Supplementary file 3 — Supplementary Material 3 [file 12909_2024_5608_MOESM3_ESM.pdf]

Supplement 3 Results of the subjective self-assessment and theory tests at time points T1 and T2

	T1<br>Mean (SD) in %	T2<br>Mean (SD) in %	p-value	Effectsize r
**Subjective competencies (self-assessment)**				
Total score subjective competence assessment	4.5 (1.0)	4.7 (0.7)	0.10	0.22
Visual perception	4.8 (1.2)	4.8 (1.0)	0.96	0.01
Spatial ability	4.4 (1.3)	4.6 (1.0)	0.20	0.17
Implementation of spatial perception in task-related movements	4.3 (1.3)	4.5 (1.0)	0.14	0.20
Knowledge of anatomical spatial relationships	4.5 (1.1)	4.9 (1.0)	0.009	0.37
**Objective competencies (Theory tests)**				
Total score theory test	47.4 (13.1)	63.5 (9.3)	< 0.001	1.34
Visual-spatial ability	61.3 (20.1)	66.9 (15.0)	0.02	0.30
Radiological cross-section image understanding and knowledge of anatomical spatial relationships	53.5 (16.2)	63.6 (15.0)	< 0.001	0.64
Understanding of radiological cross-sectional images and knowledge of anatomical spatial relationships in CT and MRI images in the abdomen	50.3 (19.0)	69.4 (14.5)	< 0.001	1.09
Understanding of radiological cross-sectional images and knowledge of anatomical spatial relationships in CT and MRI images in the neck	37.6 (24.6)	41.4 (19.5)	0.22	0.17
Theoretical ultrasound competences	34.6 (23.4)	76.0 (20.3)	< 0.001	1.84
