# Supplementary material for: Effects of undergraduate ultrasound education on cross-sectional image understanding and visual-spatial ability - a prospective study
Source: BMC Med Educ. 2024 Jun 5;24:619. doi: 10.1186/s12909-024-05608-7 (PMC11151628; doi:10.1186/s12909-024-05608-7)
Supplement: Supplementary file 4 — Supplementary Material 4 [file 12909_2024_5608_MOESM4_ESM.pdf]

**Supplement 4 Results of the analyses of possible influencing factors in relation to the results of the theory tests T1 ($Theory_{pre}$) and T2 ($Theory_{post}$).**

| **Total theory test result T1** | | | |
|---|---|---|---|
| **Factor** | **yes (mean±SD)** | **no (mean±SD)** | **p-value** |
| Previous medical experience | 48.5 ±12.6 | 44.0 ± 12.9 | 0.08 |
| Gender male | 49.8 ± 15.8 | 46.3 ± 11.5 | 0.19 |
| Previous experience in ultrasound | 52.5 ±14.5 | 46.4 ± 12.6 | 0.07 |
| Radiological knowledge | 49.5 ±15.9 | 47.2 ± 12.1 | 0.50 |
| Previous practical ultrasound experience | 56.9 ±8.9 | 46.2 ± 13.1 | <0.0001 |

| **Total theory test result T2** | | | |
|---|---|---|---|
| **Factor** | **yes (mean±SD)** | **no (mean±SD)** | **p-value** |
| Complete use of the lecture notes | 63.5±9.0 | 57.4± 22.2 | 0.77 |
| lecture notes preparation time (yes= more than 3h/week; no=less than 3h/week) | 63.2± 7.4 | 63.6± 10.2 | 0.88 |
| Dealing with other radiology topics | 67.6±5.3 | 62.8± 9.71 | 0.03 |
